# Arterial-wall origin and transient dynamics of flow- and vaso-motion activities in the awake mouse brain revealed by laser speckle contrast imaging

**DOI:** 10.1117/1.NPh.12.S2.S22804

**Published:** 2025-08-19

**Authors:** Mia Viuf Skøtt, Vladimir Matchkov, Dmitry D. Postnov

**Affiliations:** a Aarhus University, Center of Functionally Integrative Neuroscience, Aarhus, Denmark; b Aarhus University, Department of Biomedicine, Aarhus, Denmark

**Keywords:** vasomotion, flowmotion, cerebral blood flow, laser speckle contrast imaging

## Abstract

**Significance:**

Cerebral vasomotion and associated flowmotion are considered crucial for optimal brain perfusion and metabolic regulation, with reduced vasomotion being linked to cerebrovascular and neurodegenerative disorders. However, the underlying mechanisms and spatiotemporal dynamics of cerebral vasomotion are still poorly understood, underscoring the need for further research and methodological approaches for its comprehensive characterization.

**Aim:**

We aimed to develop an approach for comprehensively characterizing spatiotemporal vasomotion and flowmotion dynamics and apply it to study resting-state activity at vasomotion frequencies (0.05 to 0.25 Hz) in the awake mouse brain.

**Approach:**

Laser speckle contrast imaging (LSCI) was used to image cortical blood flow in awake mice with high spatiotemporal resolution. Pixel-wise wavelet transforms and pulsatility-based segmentation enabled the identification and quantification of transient events at the vasomotion frequency range.

**Results:**

We identified both vasomotion and flowmotion events, which displayed intermittent periods of high and low activity lasting 83.1±82.7 and 85.5±86.2  s, respectively. Temporal correlation analysis revealed the propagation of oscillatory activity from arterial walls downstream, with a delay of ∼0.33±0.58  s from the arteries to the veins.

**Conclusions:**

Our data suggest the arterial wall origin of transient vasomotion events and their consequent propagation as flowmotion in the awake mouse brain. The combined use of pulsatility imaging, wavelet analysis, and temporal clustering in the LSCI analysis provides a framework for the comprehensive characterization of vasomotion-associated blood flow dynamics.

## 1 Introduction

Blood flow in the microcirculation exhibits rhythmic fluctuations caused by multiple physiological oscillators. Vasomotion refers to spontaneous rhythmic oscillations in vascular tone and diameter of small vessels,^1^^–^^3^ leading to corresponding fluctuations in blood flow, commonly termed flowmotion.^3^ First documented over 170 years ago, vasomotion is a ubiquitous phenomenon observed in various vascular beds, including cerebral circulation.^3^ The rhythmic activity of vasomotion is inherently a property of the vascular wall itself, as, besides being triggered or modulated by neural activity,^4^^–^^6^ it maintains even in isolated arterioles.^7^ Based on the studies of short arterial segments with limited temporal resolution, vasomotion has been initially considered a global phenomenon, where all smooth muscle cells in the vascular wall are simultaneously entrained in cyclic contractions and relaxation activity.^3^^,^^7^ Moreover, any arterial segment in peripheral circulation was considered to be able to generate vasomotion of similar properties to the neighboring segment.^8^ However, recent advances in spatiotemporal imaging have proposed the dynamic nature of vasomotion, propagating along arterial vasculature, at least in cerebral circulation.^4^^,^^5^^,^^9^

Although vasomotion is widely observed across different tissues, cerebral vasomotion exhibits unique spatiotemporal characteristics. In peripheral tissues, vasomotion is often regular and sustained under stable conditions.^1^^,^^3^ In contrast, cerebral vasomotion is transient and heterogeneous,^2^^,^^10^ usually manifesting as episodic bursts rather than continuous oscillations. Vasomotion demonstrates bidirectional sensitivity to neural activity: sensory stimulation can induce transient vasomotor oscillations, whereas ongoing vasomotion activity can modify neurovascular coupling.^10^ Spatially, cerebral vasomotion is patchy, with some vessels showing robust oscillations while adjacent vessels remain quiescent.^2^ A recent *in vivo* imaging study in awake mice indicates that approximately one-third of cortical arterioles exhibit propagating vasomotion waves under resting conditions, whereas others maintain stable tone.^5^ These waves propagate along individual arterioles at speeds on the order of hundreds of micrometers per second, yet they typically lack synchronization across the entire vascular network. The neighboring arterioles may oscillate independently or even propagate waves in opposite directions.^4^ Consequently, cerebral vasomotion is best described as a “local rhythmic dance”^3^ of individual vascular segments, reflecting strong regional control in cerebrovascular perfusion regulation. This spatial heterogeneity may arise from a complex interplay among the diverse cell types within the neurovascular unit, including neurons, glial cells, smooth muscle cells, pericytes, and endothelial cells.

Heterogeneity is also reflected in the broad frequency range of oscillations in the vascular wall that is considered of a vasomotion nature.^11^ Among other oscillatory activities reflected in the vascular diameter, such as cardiac and respiratory rhythm at ∼5 to 10 Hz and 1 to 2 Hz in rodents, oscillations at a frequency range between 0.05 and 0.25 Hz are generally related to intrinsic vascular wall activities, i.e., vasomotion.^7^^,^^11^^,^^12^ The specific frequency of vasomotion is defined by a complex of intravascular and external factors, e.g., intravascular pressure, a contractile state of smooth muscle cells, endothelial cell activation, perivascular cell signaling, local metabolites, and neurovascular control.^13^

Although widely acknowledged as physiologically significant, the exact roles of vasomotion and flowmotion in brain function remain debated. Vasomotion is hypothesized to enhance local microvascular perfusion and tissue oxygenation by periodically redistributing blood flow^2^ and to facilitate interstitial fluid transport within the brain by rhythmic changes in the vessel diameter.^14^ Reduced vasomotion has been associated with impaired paravascular clearance mechanisms and implicated in neurodegenerative pathologies such as Alzheimer’s disease^2^ and cerebral amyloid angiopathy,^14^ as well as post-ischemic no-reflow conditions.^15^ This impairment may establish a vicious cycle: accumulation of amyloid-beta in vessel walls damages vascular smooth muscle cells, reducing vasomotion activity, further impairing clearance, and exacerbating amyloid deposition.^14^ Conversely, intact vasomotion may be protective in maintaining healthy cerebrovascular function. Thus, once considered merely a physiological curiosity, cerebral vasomotion is increasingly recognized as critical for brain homeostasis, with its dysfunction potentially contributing to cerebrovascular and neurodegenerative diseases.

Despite recent advances and increasing interest, the comprehensive characterization of cerebral vasomotion remains challenging. Capturing the inherently multidimensional nature of vasomotion requires simultaneous measurements of structural and perfusion dynamics over extended time periods^2^ and a large field of view.^5^ Identifying and quantifying transient vasomotion “bursts” necessitate analyses that preserve spatial and temporal resolution, as averaging over time or space would obscure their intrinsic dynamics.^16^^,^^17^ Furthermore, vasomotion is a complex multidimensional phenomenon, manifesting simultaneously across the following:

• Space: Different vessels and vascular regions may oscillate independently or in coordinated clusters.^4^^,^^5^
• Time: Oscillation intensity can vary dynamically, appearing and disappearing over time.^5^
• Frequency: Multiple frequency components or mixtures thereof may coexist within the oscillations.^7^^,^^11^

This complexity results in substantial data volumes, necessitating tailored approaches to analysis and visualization.

Here, we present a new methodological framework for characterizing cerebral vasomotion using laser speckle contrast imaging (LSCI). LSCI is an established wide-field blood flow imaging technique offering high spatial and temporal resolution,^18^^–^^20^ making it particularly suitable for capturing cerebral vasomotion.^4^^,^^17^^,^^21^^,^^22^ Our approach integrates pulsatility mapping, wavelet-based time–frequency decomposition, and temporal clustering analysis to address the abovementioned challenges. With it, we performed localization and characterization of vasomotion and flowmotion events, clearly distinguishing arterial wall–originated vasomotion activity from flow-driven oscillations elsewhere. Our findings demonstrate cerebral vasomotion’s transient and dynamic nature, revealing distinct episodes of high and low oscillatory activity. Correlation analysis further suggested arterial walls as the primary origins of vasomotion, providing evidence of its micro-arterial origin and downstream propagation throughout the cerebrovascular network.

## 2 Methods

### 2.1 Ethical Approval Declarations

All experimental protocols were approved by the Danish National Animals Experiments Inspectorate (Permit No. 2022-15-0201-01188) and conducted according to the respective guidelines and the Directive 2010/63/EU of the European Parliament on the protection of animals used for scientific purposes.

### 2.2 Animal Preparation

Male C57BL/6JRj mice (n=6, Janvier, Denmark), aged 18 weeks, were used for the study. Animals were allowed to acclimatize in their home cages for at least 7 days before undergoing surgical installation of a chronic cranial window over the left barrel cortex. We only provide a brief overview of the surgical procedure, as it was described in detail before.^23^ Mice were anaesthetized with 1.5% to 3% isoflurane mixed with oxygen, placed on a temperature-controlled heating pad (37°C), and received intraperitoneal injections of analgesics, anti-inflammatory drugs, antibiotics, and corticosteroids. Following hair removal and local anaesthesia, the scalp was excised, and the skull was cleaned and gently roughened. Then, a craniectomy (Ø=4  mm) was performed. After bone debris was removed and bleeding, if any occurred, was controlled, a glass window (Ø=4  mm) was placed over the dura mater. A metal head plate was then affixed to the skull using dental cement. Postoperative recovery lasted at least 10 days, with daily monitoring and provision of soft food. Analgesia and antibiotics were provided during the first 5 days of recovery. After recovery, mice underwent habituation to awake imaging conditions through daily restraint periods ranging from 15 min to 2 h over 10 days. Sweetened condensed milk was used as a reward during habituation. Animals were maintained under controlled housing conditions (12 h light/dark cycle, temperature 22°C to 24°C, and humidity ∼55%), with *ad libitum* access to food and water.

### 2.3 Laser Speckle Contrast Imaging

Before imaging, mice were briefly anaesthetized (3% isoflurane in 0.8  L/min oxygen) for 3 min and then transferred to the imaging stage. Animals recovered from initial anaesthesia for at least 15 min, during which system alignment and image focusing were performed. Imaging was conducted in awake animals using a custom-built LSCI system. Briefly, we implemented coaxial illumination using a polarizing beamsplitter cube and a highly coherent, volume-holographic grating-stabilized laser diode (785 nm, Thorlabs FPV785P, Newton, New Jersey, United States) coupled to a polarization-maintaining fiber. The emitted laser beam was collimated, polarization-adjusted, and directed through an adjustable Galilean telescope and steering mirror to uniformly illuminate the cortical surface via an infinity-corrected objective (Leica 2.5, N Plan, NA = 0.7). Backscattered light from the illuminated cortical region was collected by the same objective, transmitted through the beamsplitter cube, and imaged onto a CMOS camera (Basler aca2040-90um NIR, pixel size: $5.5\times 5.5\;\mu m^{2}$) using a corresponding tube lens (TTL200-B). Illumination intensity was adjusted using a neutral-density filter to achieve ∼35% of the camera’s saturation.^20^ Each recording session lasted 600 s with an acquisition rate of 194 frames per second, an exposure time of 5000  μs, and a field of view of 1024×512  pixels (∼0.5×1  mm), generating ∼58.2  GB of data per session.

### 2.4 Data Analysis

For each recording, the blood flow index (BFI) was calculated from spatial laser speckle contrast according to

$$\mathrm{BFI}=\frac{1}{K^{2}}=(\frac{\left\langle I\right\rangle }{\sigma }{)}^{2},$$ (1)

where K, ⟨I⟩, and σ denote contrast, mean, and standard deviation computed over the intensity in a 5×5  pixel neighborhood, respectively. To reduce memory requirements and allow further analysis, we decimated the BFI data in time to 4 frames per second, sufficient for studying oscillations with frequencies up to 2 Hz, including the vasomotion-driven events.

Pixel-wise continuous wavelet transforms based on Morse wavelets were applied to the entire BFI time series to characterize vasomotion dynamics in spatial, temporal, and frequency domains. For every pixel (i.e., its BFI time course), we used MATLAB’s cwt function (Morse mother wavelet, 0.01 to 1 Hz) to obtain a complex two-dimensional (2D) matrix of coefficients (frequency × time). Taking the absolute value yielded wavelet amplitudes, which were used in consequent analysis. Despite temporal decimation, such an approach generates large, ∼700  GB, four-dimensional datasets (1024×512  pixels, 84 frequency bands, and 2081 time points), impractical for direct analysis. Therefore, we employed pulsatility analysis^23^ to segment and simplify these wavelet-derived datasets spatially. Specifically, the pulsatility index (PI) was calculated as

$$\mathrm{PI}=\frac{{\max(\mathrm{BFI}}_{\text{cycle}})-{\min(\mathrm{BFI}}_{\text{cycle}})}{\left\langle {\mathrm{BFI}}_{\text{cycle}}\right\rangle },$$ (2)

where ${\mathrm{BFI}}_{\text{cycle}}$ represents the blood flow index corresponding to the average cardiac cycle and is calculated separately, without temporal decimation.^23^ Using combined PI maps and averaged BFI images, we automatically identified and masked pixels corresponding to the arteries and veins and their respective vascular walls and parenchyma. Parenchymal pixels were further subdivided into 16 equally sized bins based on their PI values. This procedure resulted in 20 spatially distinct regions for each recording, with region 1 representing arteries, region 2 arterial walls, regions 3 to 18 parenchyma, region 19 venular walls, and region 20 veins. Wavelet amplitudes, calculated for every pixel within these predefined regions, were averaged, therefore converting 2D space into a single ordered dimension along the microvascular network and enabling effective temporal and frequency-domain analyses. Time-averaged wavelet amplitude maps were computed to provide images of vasomotion spatial features.

Temporal clustering was performed to detect episodes of pronounced vasomotion activity, utilizing a modified K-means algorithm to categorize time series into distinct “flare” (high amplitude) and “silence” (low amplitude) periods. Lastly, temporal correlation analysis was conducted among different pixel regions using a sliding 60-s window to assess the propagating nature of vasomotion. This analysis aimed to identify regions possibly acting as the origin of vasomotion and flowmotion activity and to quantify delays in propagation across the vascular network.

## 3 Results

### 3.1 Spatial Distribution and Dynamics of Vasomotion and Flowmotion

**Fig. 1:**
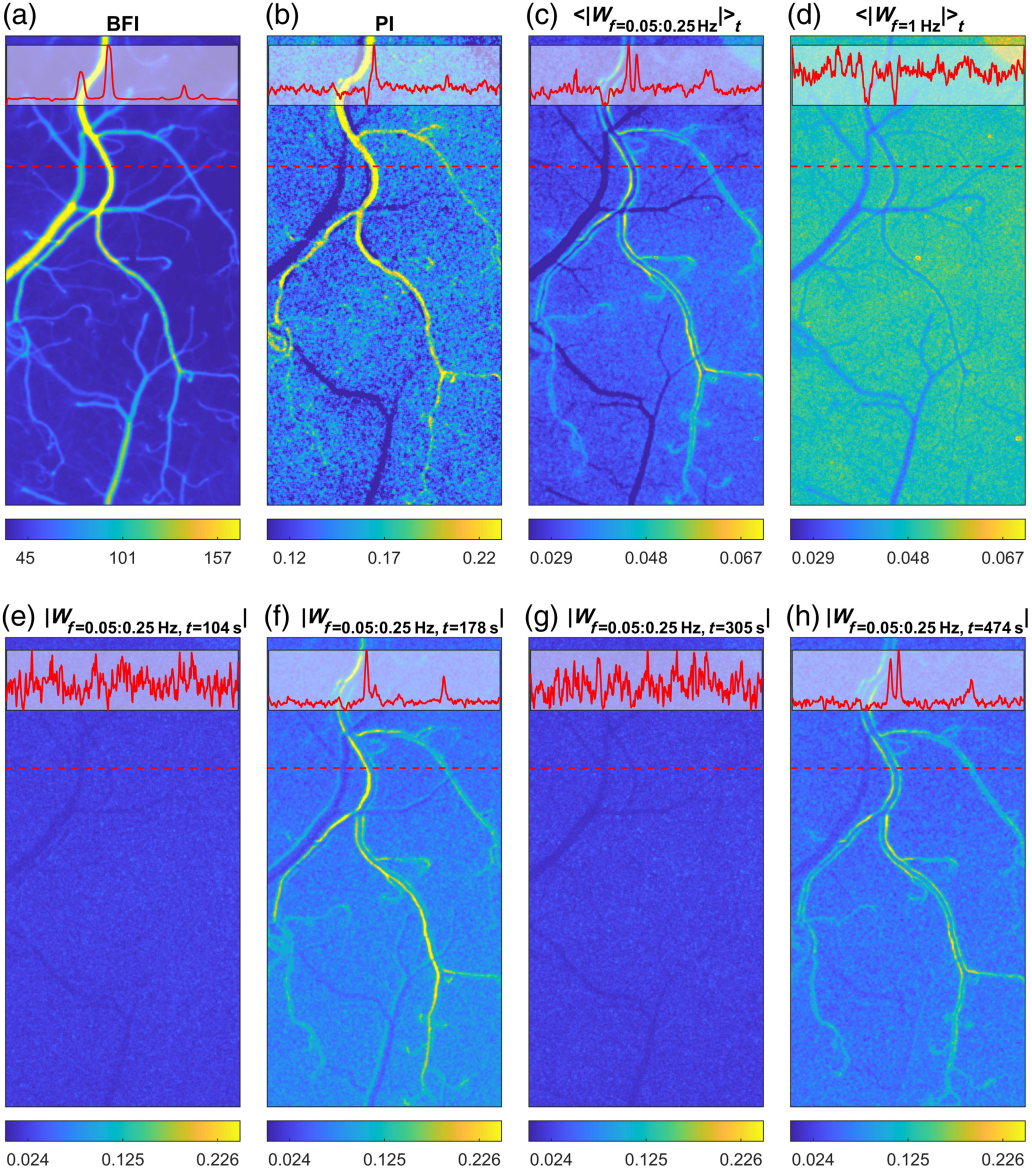
Spatial and temporal dynamics of cerebral blood flow as measured by LSCI. (a) Average BFI map. Here, and in the rest of the panels, the subplot at the top of the image reflects the cross-section values (dashed red line). (b) Corresponding PI map. (c) and (d) Time-averaged (over the entire recording duration) wavelet amplitude maps illustrating oscillatory activity at vasomotion frequencies (0.05 to 0.25 Hz) and other oscillatory activity at 1 Hz. Note that at the vasomotion frequencies, the activity is the most pronounced in the arterial walls and small arteries, less in the parenchyma, and least in the veins. (e)–(h) Representative temporal snapshots (at 104th, 178th, 305th, and 474th seconds of the recording, respectively) of wavelet amplitude at vasomotion frequencies, highlighting transient patterns and the dynamic appearance and disappearance of vasomotion events.

Laser speckle contrast imaging revealed distinct blood flow patterns along the cerebral microvascular network. Consistent with previous findings,^23^ the average BFI increased with vessel diameter on both arterial and venular sides [Fig. 1(a)]. Conversely, the pulsatility index progressively decreased along the vascular network, reaching its lowest values in the veins [Fig. 1(b)]. This inverse relationship between BFI and PI enabled automated spatial segmentation, as described in Sec. 2.

Wavelet amplitude in the vasomotion frequency range (0.05 to 0.25 Hz) averaged over the entire recording duration ($\langle |W_{f=0.05∶0.25\;\mathrm{Hz}}|\rangle_{t}$) demonstrated prominent oscillatory activity along the arterial walls [Fig. 1(c)]. The observed activity is less pronounced in the parenchyma and is weakest within veins. Higher frequency oscillations (around 1 Hz, $\langle |W_{f=1\;\mathrm{Hz}}|\rangle_{t}$), attributed primarily to noise and respiratory artefacts, showed no clear distinction between the arteries and the veins and were distributed more uniformly throughout the microvasculature [Fig. 1(d)]. Temporal snapshots of wavelet amplitude further emphasized the transient and dynamic nature of the vasomotion and flowmotion activity, highlighting evolving spatial patterns over short time scales [Figs. 1(e)–1(h)].

**Fig. 2:**
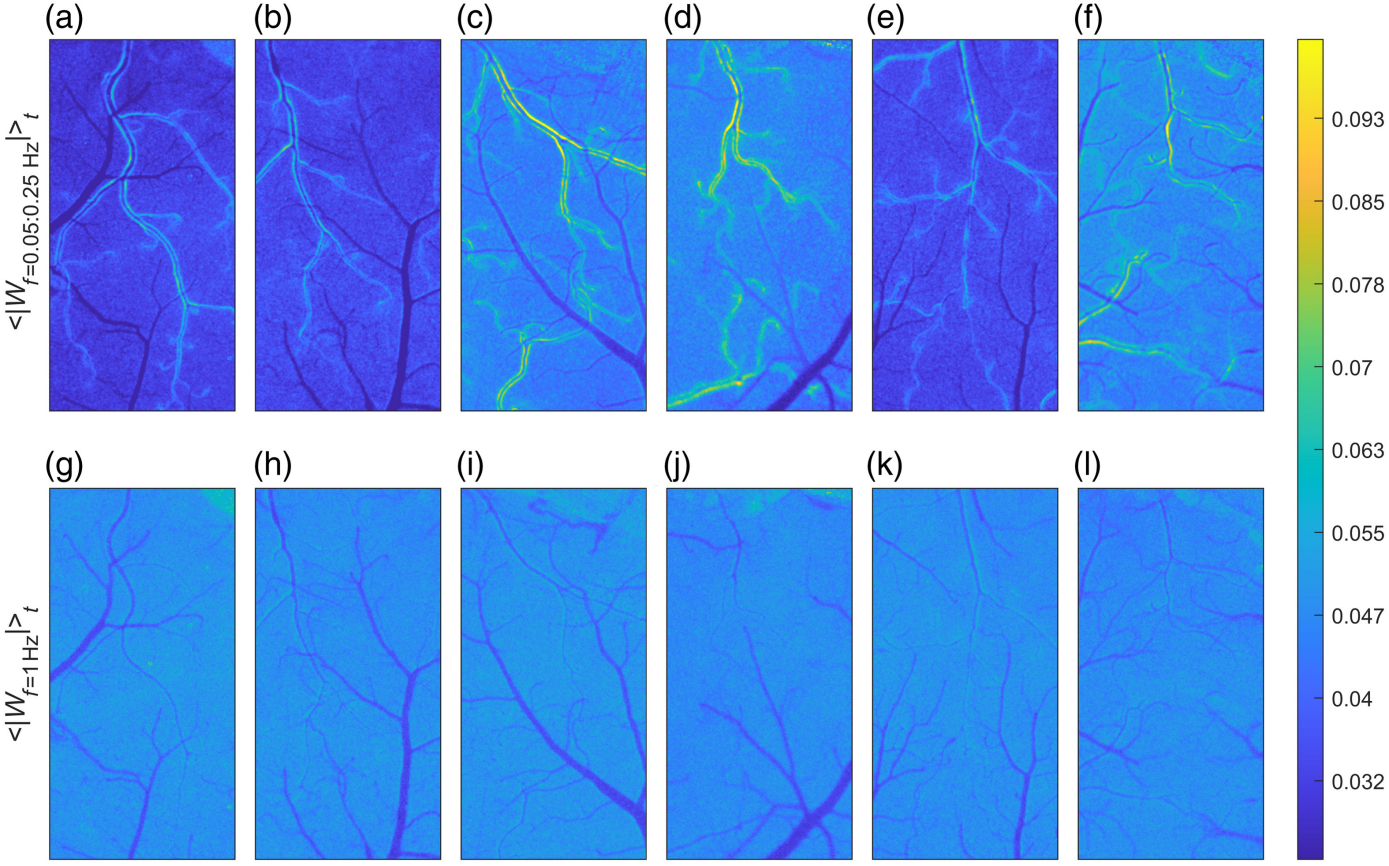
Spatial distribution of wavelet amplitudes is consistent across animals, with arterial walls demonstrating the most pronounced activity within the vasomotion frequency range. Panels (a)–(f) represent time-averaged wavelet amplitude maps at vasomotion frequencies (0.05 to 0.25 Hz), and panels (g)–(l) correspond to 1 Hz frequency. Each column pair [e.g., panels (a) + (g)] corresponds to one individual animal.

The observed spatial distribution of vasomotion-related wavelet amplitudes was consistent across all animals. The arterial walls uniformly exhibited the highest amplitude increases at vasomotion frequencies, whereas at higher frequencies, the arteries and veins remained indistinguishable (Fig. 2).

**Fig. 3:**
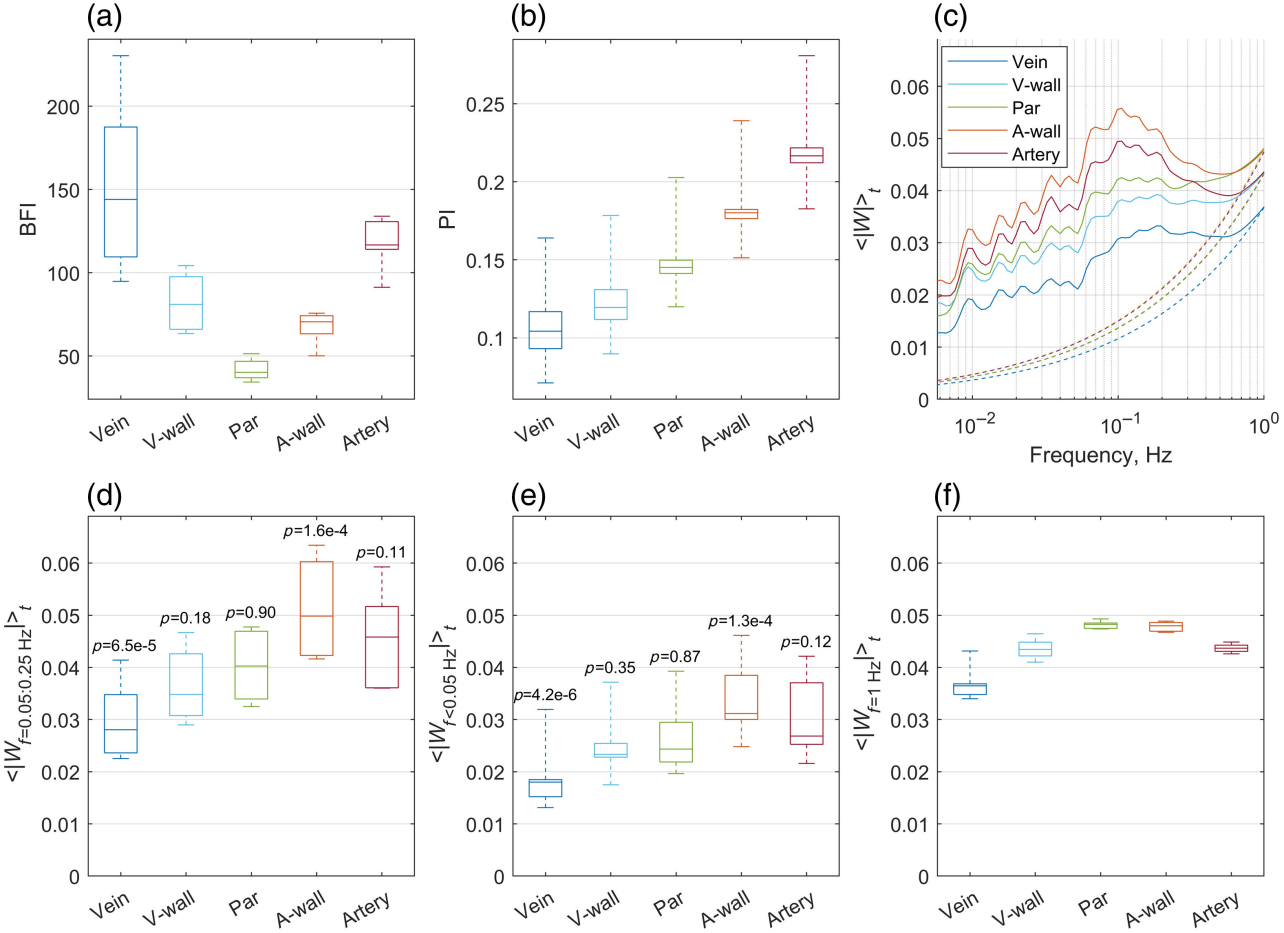
Quantitative characterization of vasomotion and flowmotion across the microvascular network. (a) Boxplot summarizing the average BFI across predefined regions in all animals. (b) Boxplot summarizing PI across predefined regions in all animals. (c) Time- and animal-averaged wavelet amplitudes (solid lines) for distinct vascular regions with estimated noise levels (dashed lines). The following peaks can be identified within the 0.05:025 Hz range: 0.07, 0.106, 0.13, and 0.184 Hz, with the overall peak amplitude reached in the arterial wall region at 0.106 Hz. (d) Boxplot of average wavelet amplitude at vasomotion frequencies (0.05 to 0.25 Hz). The statistical significance values denoted in the plot were calculated based on the linear mixed model test, where wavelet amplitude is the response value, and the target region is the predictor compared with the data pulled from other regions. The comparison highlights the arterial walls and veins as strong predictors of wavelet amplitude, with $p=1.6\times 10^{-4}$ and $p=6.5\times 10^{-5}$, respectively. (e) Boxplot of average wavelet amplitude at frequencies below 0.05 Hz. The arterial walls and veins are identified as strong predictors of wavelet amplitude, with $p=1.3\times 10^{-4}$ and $p=4.2\times 10^{-6}$, respectively. (f) Boxplot of average wavelet amplitude at 1 Hz frequency. Unlike vasomotion-associated and slow oscillatory activities, where wavelet amplitudes signify the presence of rhythmic vessel diameter changes, no prominent activity is observed in the arterial wall at 1 Hz.

Figure 3 quantifies these findings by analyzing region-averaged data across the animals. Unlike the U-shaped distribution of BFI, which has high values in arteries and veins and lower in parenchyma [Fig. 3(a)], and the monotonic decrease of PI from arteries to veins [Fig. 3(b)], wavelet amplitude associated with vasomotion frequency range reached a clear maximum ($p=1.6\times 10^{-4}$ based on the linear mixed model test) in the arterial wall regions [Figs. 3(c) and 3(d)]. This peak indicates rhythmic oscillations in vascular diameter (vasomotion), whereas flowmotion (velocity changes without diameter variations) appeared weaker within arterial lumens and progressively decreased through the parenchyma toward the venular regions and veins. Although wavelet amplitude was elevated at the venular walls compared with the veins, it did not exceed surrounding parenchymal levels, suggesting that observed activity at venular wall regions reflects the mix of flow dynamics between parenchymal and venular regions rather than venular diameter oscillations. Slow oscillations (below 0.05 Hz, $\langle |W_{f<0.05\;\mathrm{Hz}}|\rangle_{t}$) exhibited similar spatial distributions, with the arterial walls having the highest wavelet amplitudes ($p=1.3\times 10^{-4}$) though less pronounced than in the vasomotion frequency range [Fig. 3(e)]. The pattern is gone for frequencies around 1 Hz, where the parenchyma demonstrates the highest wavelet amplitudes [Fig. 3(f)] and no increased activity is observed in the artery-associated regions.

### 3.2 Temporal Characterization and Clustering of Vasomotion and Flowmotion Events

**Fig. 4:**
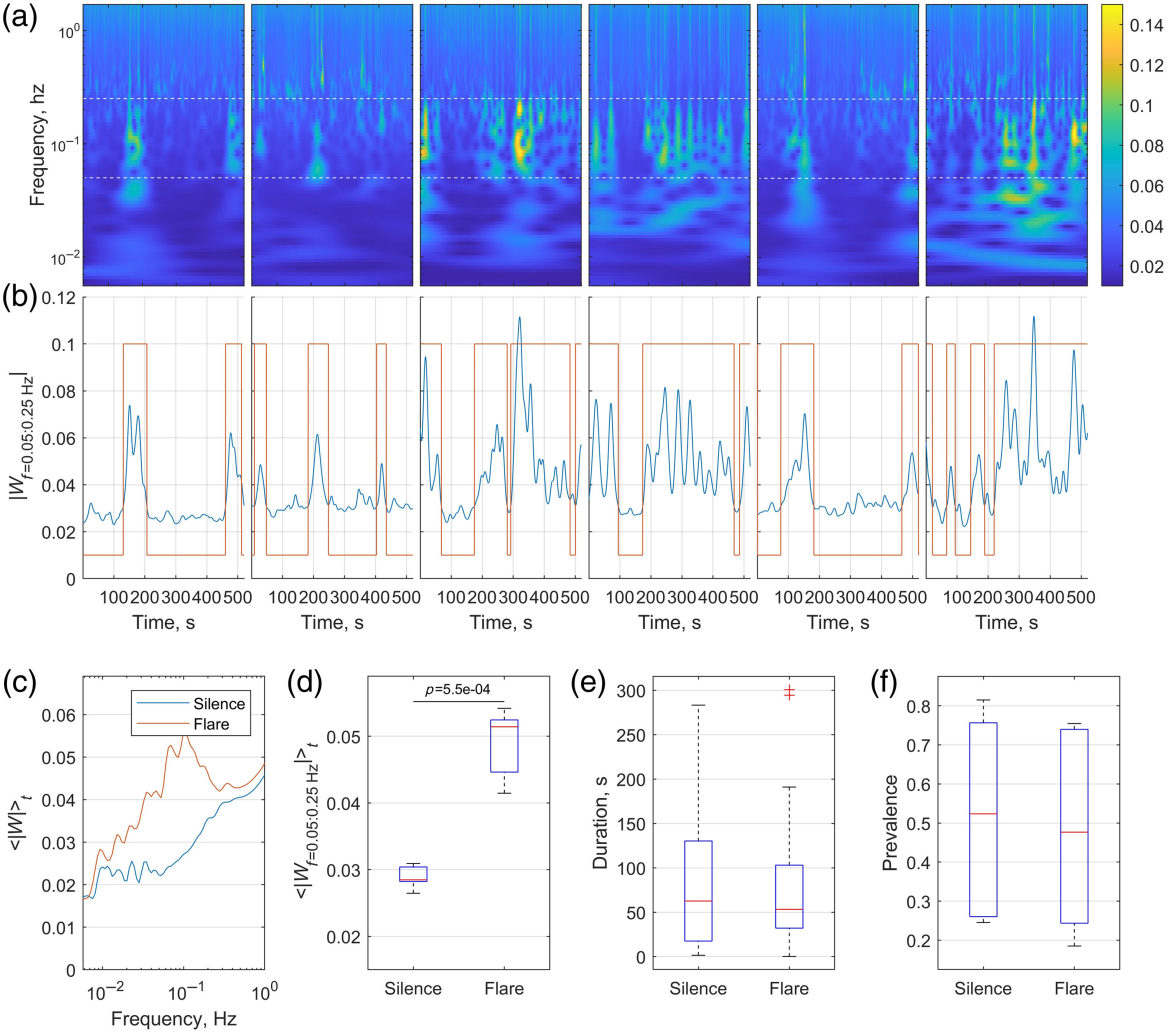
Temporal analysis and clustering of vasomotion activity. (a) Space-averaged wavelet amplitude across frequencies and time (one panel per animal). Dashed lines delineate the vasomotion frequency range (0.05 to 0.25 Hz). (b) Corresponding time series of wavelet amplitude averaged over space within the vasomotion frequency band (blue lines) and clustering-based identification of silence and flare periods indicated in orange. (c) Average wavelet amplitude spectra during silence (blue) and flare (orange) periods. Although the entire vasomotion frequency range has elevated amplitude, the two most prominent peaks can be identified at 0.07 and 0.106 Hz. (d) Boxplot comparing the average wavelet amplitude within the vasomotion frequency band between silence and flare states. The amplitudes are significantly different, as confirmed by the paired t-test ($p=5.5\times 10^{-4}$). (e) and (f) Boxplots summarize the duration and prevalence of silence and flare periods, respectively.

Analysis of temporal vasomotion dynamics revealed clearly distinguishable intervals of elevated (“flare”) and reduced (“silence”) vasomotion activity [Figs. 4(a) and 4(b)]. Applying a modified k-means clustering algorithm allowed automatic segmentation of these two distinct states based on wavelet amplitude fluctuations. Space- and frequency-averaged wavelet amplitudes during flare periods were significantly higher ($p=5.5\times 10^{-4}$) compared with silence periods, with the differences remaining consistent across animals [Figs. 4(c)–4(d)].

Flare and silence periods had similar duration—lasting 83.1±82.7 and 85.5±86.2  s, respectively—and a prevalence of 0.52±0.28 and 0.48±0.28. Considerable variability in duration and prevalence among animals has contributed to the differences observed in the average wavelet amplitude images of vasomotion activity [Figs. 2(a)–2(f)], although it did not affect the “noise-associated” wavelet amplitudes at 1 Hz [Figs. 2(g)–2(l)].

### 3.3 Propagation Patterns and Temporal Correlation of Vasomotion and Flowmotion

**Fig. 5:**
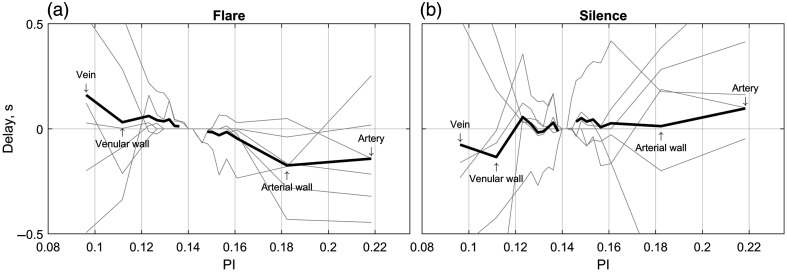
Vasomotion and flowmotion activity appear to propagate along the cerebrovascular network. Plots indicate the delay (in seconds) of maximum temporal correlation of vasomotion-associated wavelet amplitudes relative to parenchymal regions with median PI. (a) Propagation during the flare periods suggests initiation of activity at arterial walls, progressing as flowmotion through the arteries, parenchyma, and finally reaching the veins. (b) Lack of directed propagation during silence periods, highlighting the specificity of vasomotion-related signal propagation.

Local temporal correlation analysis using sliding 60-s windows suggests propagation of vasomotion and flowmotion activity along the cerebral microvascular network. On average, wavelet amplitude changes associated with vasomotion flares originated within arterial wall regions and subsequently propagated downstream, reaching the venous compartment with a delay of 0.33±0.58  s [Fig. 5(a)]. This directional propagation was exclusive to vasomotion flare periods and was absent during the silence periods, underscoring the functional specificity of this phenomenon [Fig. 5(b)].

## 4 Discussion

We employed laser speckle contrast imaging combined with wavelet-based time-frequency and pulsatility analysis to overcome longstanding methodological challenges in the characterization of cerebral vasomotion and flowmotion. Traditional imaging modalities often fail to capture the transient and spatially heterogeneous nature of these phenomena. Our approach, however, facilitated a comprehensive characterization of vasomotion-driven activity across spatial, temporal, and frequency domains.

Our results support the hypothesis that cerebral vasomotion originates predominantly from the walls of small pial arteries.^13^^,^^24^ This conclusion is substantiated by consistently elevated wavelet amplitudes observed in the arterial wall regions compared with parenchymal and venous regions [Fig. 1(c) and Figs. 3(c)–3(e)]. Elevated amplitudes at vessel edges reflect rhythmic oscillations in the vessel diameter, which lead to periodic transitions of pixels between the vessel lumen and the parenchyma, thus producing pronounced oscillations in the blood flow index. Interestingly, vasomotion events were not confined to individual arteries but encompassed multiple vessels, supporting the hypothesis of coordinated oscillations.^6^ Also, although we generalize the vasomotion and flowmotion frequency range as 0.05 to 0.25 Hz, the data suggest several peaks which can be identified within it: at 0.07, 0.106, 0.13, and 0.184 Hz, with the overall peak amplitude reached in the arterial wall region at 0.106 Hz [Fig. 3(c)]. The temporal correlation analysis further reinforced this conclusion, suggesting that vasomotion oscillations in the BFI data originate from the arterial walls and propagate as flowmotion into the arterial lumens and downstream to the parenchyma and veins [Fig. 5(a)]. We detected a delay of 0.33±0.58  s between vasomotion oscillations in the arterial walls and the appearance of flow-driven oscillations in the veins. Although LSCI does not allow direct estimation of the distance along the vascular network, even a minimal artery-to-vein separation of a few millimeters places the apparent flowmotion speed order of magnitude above the propagating vasomotion waves reported earlier.^5^ This discrepancy is expected, as flowmotion propagation, among other factors, is governed by the pressure wave propagation, which traverses the cortical microvasculature in 10 to 20 ms.^25^ However, we should note that the decimation in the initial processing steps limits the temporal resolution of the analysis, making it insufficient to quantify pressure wave propagation and likely increasing the error in the presented delay measurements. Therefore, spatial propagation and the coordination mechanisms underlying these oscillations remain an open question. For future studies, we suggest scaling the presented methodological approach to the even larger field of view, preferably covering both hemispheres and higher framerates (or no decimation) to address these questions.

Temporal clustering analysis demonstrated that cerebral vasomotion dynamics can be characterized by alternating intervals of pronounced (“flare”) and reduced (“silence”) oscillatory activity. Both flare and silence periods exhibited a duration of 83.1±82.7 and 85.5±86.2  s respectively, and a prevalence of 0.52±0.28 and 0.48±0.28 [Figs. 4(f) and 4(g)]. High variability in both duration and prevalence, but not the wavelet amplitude, observed within and among animals, underscores the transient and irregular nature of cerebral vasomotion. Consequently, prolonged recordings (e.g., 30 min or longer) would be advantageous to comprehensively characterize these temporal dynamics, minimizing potential biases resulting from incomplete or uneven sampling of flare and silence cycles. Intriguingly, the near-equality of the mean and standard deviation of flare durations points to an approximately exponential distribution because an exponential law is the only continuous distribution of positive numbers whose first two moments are identical. Confirming with a larger sample and longer recordings would imply that vasomotion flares follow a memory-less (Poisson) process^26^ and will suggest an association of vasomotion flares with other processes in the brain governed by similar stochastic dynamics, including the exponential inter-spike interval distribution of cortical neurons^27^^,^^28^ and the exponential open-time distribution of single ion channels.^29^

Our findings underscore the suitability of LSCI as a powerful imaging modality for studying cerebral vasomotion and flowmotion, particularly when coupled with appropriate data analysis strategies. Integrating the pulsatility index was particularly advantageous, effectively transforming a complex, multidimensional vascular space into a simplified one-dimensional continuum along the vascular tree (Fig. 3). In addition, the application of time-resolved wavelet analysis preserved transient oscillatory features that would typically be obscured by conventional approaches, such as the fast Fourier transform, which often averages signals over extended periods. Furthermore, temporal clustering into flare and silence periods significantly enhanced the accuracy and interpretability of our findings.

An important limitation of the current study, inherent to LSCI in general,^18^^,^^19^^,^^30^ is that blood flow changes originating from deeper vascular structures are detected only indirectly, manifesting as averaged signals within the parenchyma. Therefore, it remains unclear at which vascular level the vasomotion activity observed in larger arteries is terminated. Future studies combining LSCI with imaging modalities capable of resolving deeper cerebral vasculature, such as multiphoton microscopy, must fully delineate vasomotion’s spatial extent along the deep arterial network.

Overall, our results elucidate the arterial wall origin and propagation dynamics of cerebral vasomotion, providing crucial methodological insights for its accurate characterization through LSCI.

## 5 Conclusion

Our study demonstrates that laser speckle contrast imaging, combined with pulsatility segmentation and wavelet-based time-frequency analysis, effectively captures cerebral vasomotion and associated flowmotion dynamics. We provide robust evidence that vasomotion originates primarily from the arterial walls, characterized by significantly higher wavelet amplitudes compared with parenchymal and venous regions, and propagates downstream as flowmotion, reaching the venous compartment after a delay of 0.33±0.58  s.

Temporal clustering analysis revealed distinct intermittent periods of pronounced (“flare”) and reduced (“silence”) vasomotion activity, lasting for 83.1±82.7 and 85.5±86.2  s, respectively. The integration of pulsatility indexing, wavelet transforms, and temporal clustering successfully simplified the inherently complex and multidimensional cerebrovascular dynamics, preserving critical transient events otherwise obscured by traditional analysis methods.

These methodological advancements substantially enhance our ability to characterize vasomotion comprehensively, laying a solid foundation for further investigations into cerebrovascular regulation and its implications in cerebrovascular and neurodegenerative diseases.

## Data Availability

Data and code underlying the results presented in this paper may be obtained from the authors upon request.
